# LINC02389/miR-7-5p Regulated Cisplatin Resistance of Non-Small-Cell Lung Cancer via Promoting Oxidative Stress

**DOI:** 10.1155/2022/6100176

**Published:** 2022-10-19

**Authors:** Peng Ma, Wen Han, Cunying Meng, Xiaohong Tan, Pengfei Liu, Lei Dong

**Affiliations:** ^1^Department of Gastroenterology, Second Affiliated Hospital of Xi'an Jiaotong University, Xi'an, 710061 Shaanxi Province, China; ^2^Department of Gastroenterology, Affiliated Hospital of Yan'an University, Yan'an, 716000 Shaanxi Province, China

## Abstract

**Background:**

Non-small-cell lung cancer (NSCLC) is one of the most common malignancies worldwide, and cisplatin-based chemotherapy is the main treatment for NSCLC. However, cisplatin resistance of NSCLC cells is a major challenge for NSCLC treatment.

**Materials and Methods:**

qRT-PCR and Western blot were performed to detect the expression of LINC02389 and miR-7-5p in NSCLC tissues and cell lines. Cell counting kit-8 (CCK-8) assay and flow cytometry assay were applied to exam cell proliferation and apoptosis rate of NSCLC cells. The interaction between LINC02389 and miR-7-5p was verified by dual luciferase reporter gene assay, RNA pull-down assay, and RNA immunoprecipitation (RIP) assay. Additionally, cisplatin-resistant NSCLC cells were generated to assess the biological function of LINC02389 and miR-7-5p in cisplatin resistance of NSCLC.

**Results:**

LINC02389 was highly expressed in NSCLC tissues and was correlated with poor prognosis of NSCLC patients. Knockdown of LINC02389 inhibited cell proliferation and promoted cell apoptosis of NSCLC, whereas miR-7-5p knockdown exerted the opposite effects. Moreover, LINC02389 negatively regulated the expression of miR-7-5p. In addition, LINC02389 was overexpressed, yet miR-7-5p was downregulated in cisplatin-resistant NSCLC cells compared with their parental cells. Moreover, oxidative stress biomarkers were overexpressed in cisplatin-resistant cells and were regulated by LINC02389. Besides, LINC02389 could reverse the inhibitory effect of cisplatin on NSCLC cells, which was partially reversed by attenuating the expression of miR-7-5p.

**Conclusion:**

Our research firstly demonstrated that lncRNA LINC02389 acted as an oncogene to promote progression, oxidative stress, and cisplatin resistance through sponging miR-7-5p and may provide therapeutic targets for NSCLC.

## 1. Introduction

Lung cancer is the cancer with the highest mortality and morbidity worldwide, causing a huge medical burden for cancer treatment worldwide [1, 2]. However, deteriorating air conditions and a booming tobacco economy have placed people at risk of a variety of lung cancer causes and different types of cancer [3, 4]. Among them, non-small-cell lung cancer (NSCLC) is the most common lung cancer. For perioperative or unresectable NSCLC, adjuvant therapies such as chemotherapy, radiotherapy, and targeted therapy play an important role [5, 6]. Among them, cisplatin-based chemotherapy has become the first-line treatment for unresectable NSCLC. Interestingly, cisplatin exerts its anticancer effects by damaging nuclear DNA [7, 8], but DNA repair mechanisms will facilitate the rapid acquisition of cisplatin resistance in cancer cells [9]. Therefore, cisplatin resistance has become a difficult problem in current clinical treatment, and it is necessary to further explore how to reverse this drug resistance.

Recently, lncRNAs (long noncoding RNA) have been identified to be involved in mediating cisplatin resistance [10]. lncRNA is a group of RNA which does not encode proteins but plays a vital role in epigenetic regulation and modification [11]. lncRNA always exerts their functions by interacting with mRNA, miRNA, proteins, and even DNA [12]. Many biochemistry intracellular processes such as epithelia mesenchymal transition (EMT) and oxidative stress are regulated by lncRNA through its upstream effects [13]. In NSCLC initiation and progression, lncRNA MALAT1 is able to cause chemoresistance by the miR-197-3p/p120 catenin axis [14]. Otherwise, lncRNA functions in a way called compete endogenous RNA (ceRNA). lncRNA PTAR can sponge miRNA101 like a sponge that results in the miRNA downregulation and promote NSCLC cell proliferation, migration, and invasion [15]. LINC02389 is reported to be a significant prognostic factor in lung squamous cancer in a genome-wide analysis [16]. However, the function of LINC02389 in NSCLC remains buried.

miRNA exhibits inhibitive effects in gene transcription initiation and the stability and translation of mRNA in recent studies [17]. As a member of miRNA family, miR-7 plays a crucial role in posttranscriptional modification. Moreover, substantial studies of miR-7-5p have revealed that miR-7-5p is a tumor suppressor in hepatocellular carcinoma, color rectal cancer, breast cancer, and glioblastoma. Downregulation of miR-7-5p causes OGT overexpression in colorectal cancer resulting in disease progression [18]. It is also reported in colorectal cancer that miR-7-5p is negatively regulated by ZFAS1 inducing cancer cell progression [19]. miR-7-5p is also involved in glioma tumorigenesis via the miR-7-5p/EGFR/PI3K/AKT/c-MYC feedback loop [20]. Intriguingly, miR-7-5p downregulates KLF4 in colorectal cancer as well as in esophageal cancer, but this biological downregulation inhibits tumorigenesis of esophagus cancer [21, 22]. As discussed before, miR-7-5p is negatively related both in gastric cancer and glioblastoma coincidentally [23, 24]. In addition, miR-7-5p is determined to be related with tumor recurrence and metastasis in a cohort study [25]. Considering the cancer cell chemoresistance, this miRNA promotes resistance of cervical cancer cells but attenuates doxorubicin resistance of small-cell lung cancer cells [26, 27]. Among the NSCLC, Li et al. found that miR-7-5p induces apoptosis, cell growth inhibition, and cell cycle arrest via regulating PAK2 [28]. Generally, miR-7-5p has already been widely reported in cancer. In this study, we prove a novel mechanism of miR-7-5p regulation in NSCLC. The underlying mechanisms in LINC02389/miR-7-5p-regulated cisplatin resistance are still obscure. Herein, we conducted experiments in vitro to analyze the role of LINC02389/miR-7-5p alteration which will provide a novelty in NSCLC treatment.

## 2. Method

### 2.1. Patients

From 2016 to 2018, 257 patients admitted to the Second Affiliated Hospital of Xi'an Jiaotong University were recruited into the experiment after being diagnosed with NSCLC by imaging and histopathological examinations. All patients have been fully informed and signed the written consent. Cancer and adjacent normal tissues were obtained during the surgery. Our study meets the requirements of declaration of Helsinki and was approved by the ethics committee of the Second Affiliated Hospital of Xi'an Jiaotong University (no. 2015-ms-45).

### 2.2. RNA Sequencing to Identify Differentially Expressed lncRNA

Five cisplatin-based chemotherapy-resistant and 5 cisplatin-based chemotherapy-sensitive patients' cancer and adjacent tissues were collected and frozen for RNA sequencing to identify the differentially expressed lncRNA between resistant and sensitive cancer tissues.

### 2.3. Cell Culture and Transfection

A549, HCC827, and MRC-5 cell lines were obtained from American type culture collection (ATCC, Genetimes, Shanghai, China). A549 was cultivated with Ham's F-12K (Kaighn's) medium and HCC827 was treated with RPMI 1640 medium. MRC-5 cells were cultured with DMEM. All the medium was supplemented with 10% FBS and 1% penicillin and streptomycin. Cells were incubated at 37°C with 5% CO_2_ and proper humidity.

To establish the LINC02389 and miR-7-5p knockdown and miR-7-5p overexpression cell model, lentivirus was constructed and generated by GeneChem (Shanghai, China). Cells were seeded in a 6-well plate before the day of transfection and cultivated to 50-80% confluency. Afterwards, treat the lentivirus with prepared cells following the introductions and amplify the stable transfected cells for couple of days. The efficiency could be visualized by fluorescence microscope. qRT-PCR was performed to detect the expression level.

### 2.4. qRT-PCR

Each group of cells was washed with PBS three times and extracted with TRIzol reagent. Isolate the total RNA from the cytoplasm and nucleus by centrifugation. The Advantage RT-for-PCR Kit (TaKaRa, Japan) was applied for reverse transcription and PCR assay. Reversely transcribe the RNA into cDNA following the instruction. The thermal cycler was programmed and heated already before the experiment. Place each group of template RNA into the system and set up the reaction. Collect the relative expression level of each group and analyze the final data subsequently.

### 2.5. Western Blot

Each group of cells was washed with PBS three times and extracted with RIPA supplemented with protease inhibitor and phosphatase inhibitor on ice for 10 mins. Vortex and centrifuge the solution at 4°C and pipette the supernatant for BCA quantitative analysis to identify the protein concentration. BCA kits were provided by Beyotime Biotechnology (Shanghai, China). Each protein sample was supplemented with 5x loading buffer and boiled for 10 mins. Then, samples were electrophoretically separated on sodium dodecyl sulfate-polyacrylamide gel (SDS-PAGE) and transferred onto polyvinylidene fluoride (PVDF) membrane via semidry assay afterwards. Block the membrane in 5% skim milk for 1 h and wash with TBST slightly. Incubate the membrane with antibody against H3K27ac in tube at 4°C and shake overnight. Histone H3 was set as internal control. Wash the membrane with TBST three times for 10 mins each and then incubate the corresponding secondary antibody with H3K27ac and Histone H3 for 1-2 h at room temperature. Eventually, wash the membrane three times with TBST for 10 mins each before detection.

### 2.6. ChIP

The ChIP assay was performed with the Chromatin Immunoprecipitation (ChIP) Assay Kit (Beyotime, China). Different groups of cells were crosslinked with 1% formaldehyde for about 10 mins at room temperature and quenched with glycine. After ultrasonication, the cell lysates were incubated with primary antibody anti-H3K27ac overnight. The immunoprecipitated DNA fragments were subjected to qPCR analysis with LINC02389-specific primers described before.

### 2.7. Dual Luciferase Reporter Gene Assay

For the miR-7-5p target LINC02389 dual luciferase reporter gene assay, synthesized PmirGLO-LINC02389-wt/mut vector and miR-7-5p-overexpression/NC vector were cotransfected with A549 and A549-R cells. PmirGLO-LINC02389-wt/mut vector and miR-7-5p-overexpression/NC vector were designed and synthesized by GeneChem (Shanghai, China). The luciferase activity was measured with the Dual Luciferase Assay system, and the luciferase activity was normalized via Renilla luciferase activity.

### 2.8. RNA Pull-Down Assay

RNA pull-down assay was performed to explore the internal crosstalk between LINC02389 and miR-7-5p. Magnetic beads with specific binding sites to LINC02389-wt/mut were incubated with cells overnight, followed by elute and purification. The resultant was subjected to qRT-PCR to detect the miR-7-5p expression.

### 2.9. ROS

The ROS/superoxide detection assay kit (cell-based) (Abcam, UK, ab139476) was utilized to detect the ROS level in each group. Seed the cells into polystyrene tissue culture plates one day before the experiment and make sure a 50-70% confluency on the day of experiment. Then, follow the instruction of the assay kit and handle the different group of cells properly. Detect the results via microplate reader and collect the data for further analysis.

### 2.10. ELISA

To detect the MDA and GSH and SOD expressions in cells, ELISA was performed using a relevant ELISA kit from Abcam (MDA, ab238537; GSH, ab193767; SOD, ab277415), following the manufacturer protocol. Specific antibody to MDA and GSH and SOD linked with enzyme was incubated with cell lysates. The activity of enzyme demonstrates the expression level of each protein.

### 2.11. Transwell

The cell migration capacity was detected by Transwell assay. Different groups of cells were seeded onto small chambers added serum-free medium place on the 12-well plate. The lower wells were loaded with specific medium supplemented with FBS. After 24 h cultivation at 37°C, remove the upper chamber and fix with paraformaldehyde and stain with crystal violet. Image the results via a microscope.

### 2.12. CCK-8

Cell counting kit 8 (CCK-8, Abcam, Shanghai, China) was utilized to detect the proliferation rate and IC50 of A549 and A549-R cells. For both experiment processes, cells were plated into a 96-well plate and cultivated to 50% confluency approximately. Add CCK-8 reagent to each well and incubate for a proper time following the instructions, and the cell proliferation rate curves should be plotted by measuring the optimal density (OD) at 450 nm. Besides, for the IC50 detection, different concentrations of cisplatin should be added to each well to form a concentration gradient.

### 2.13. Cell Apoptosis Assay

Caspase 3/7 detection reagent (GeneChem, Shanghai, China) was applied to detect the apoptosis rate. Briefly speaking, add diluted reagent to cells and incubate 30 mins, and then, measure the fluorescence at 502-530 nm approximately.

### 2.14. Statistics

All the results were replicated at least three times, and the most representative figure was demonstrated. All data are demonstrated as the mean ± S.D. Student's *t*-test was performed to identify the difference between two independent samples, and the difference among the groups was determined by two-way ANOVA using GraphPad Prism 8.2 (GraphPad Prism Software, San Diego, CA, USA). Highlighted in the pictures with a horizontal line means *P* value of <0.05 was considered statistically significant.

## 3. Result

### 3.1. LINC02389 Expression Profile in NSCLC Tissues

**Figure 1:**
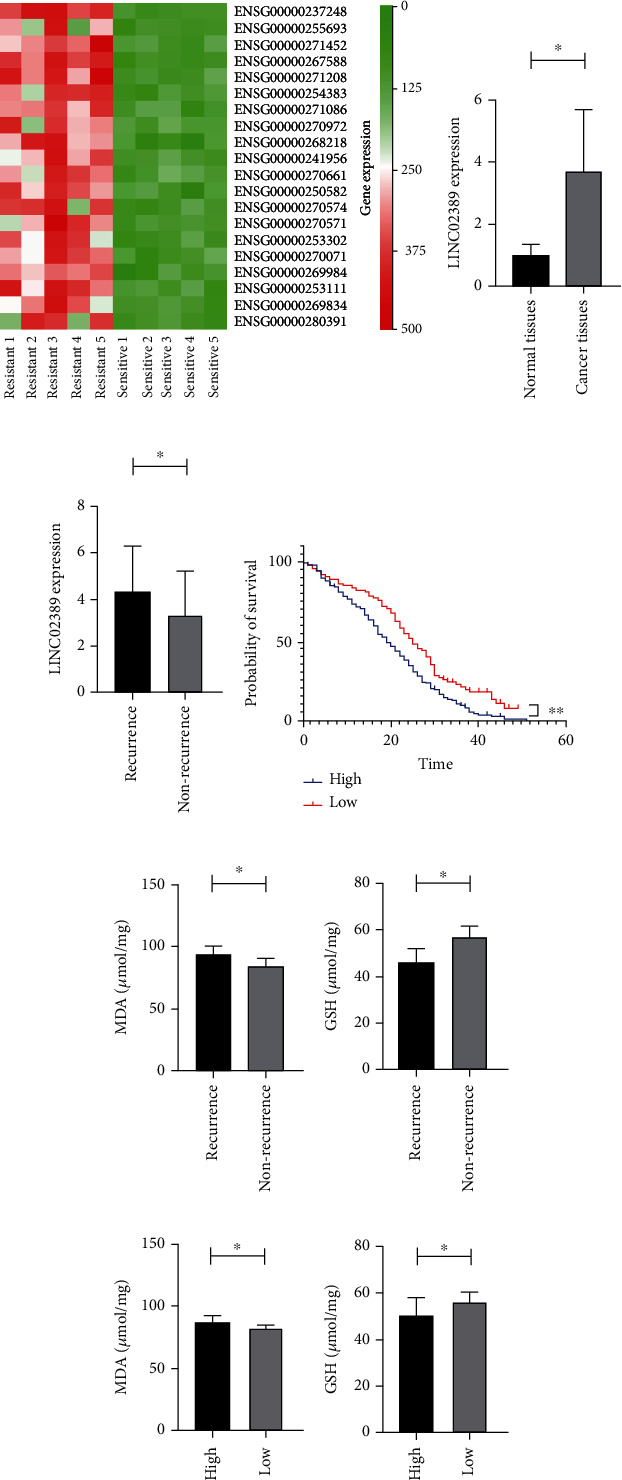
(a) Heat map to identify differentially expressed lncRNAs between cisplatin-resistant and cisplatin-sensitive patients. (b) LINC02389 expression in non-small-cell lung cancer patients and adjacent normal tissues. (c) LINC02389 expression in recurrent and nonrecurrent non-small-cell lung cancer patients. (d) Survival analysis for LINC02389 high and low expressed non-small-cell lung cancer patients. (e, f) MDA and GSH expressions in recurrent and nonrecurrent non-small-cell lung cancer patients. (g, h) MDA and GSH expressions in LINC02389 high and low expressed non-small-cell lung cancer patients.

LINC02389 was identified by RNA sequence; heat map for top 20 differentially expressed genes between cisplatin-based chemotherapy-resistant (*n* = 5) and chemotherapy-sensitive groups (*n* = 5) are shown in Figure 1(a). In 257 NSCLC patients, LINC02389 was overexpressed in cancer tissues compared with normal tissues (Figure 1(b)). Survival analysis showed that LINC02389 correlated with poor prognosis in 257 NSCLC patients (Figure 1(d)).

In addition, in 93 local recurrent patients, we observed higher expression of LINC02389, indicating its potential role in mediating cisplatin-based chemotherapy resistance (Figure 1(c)). Moreover, we have found increased MDA level in local recurrent patients (Figure 1(e)) and decreased GSH expression in local recurrent patients (Figure 1(f)), suggesting that oxidative stress plays important roles in regulating cisplatin-based chemotherapy resistance. Furthermore, we have found increased MDA level and decreased GSH expression in LINC02389 high expressed patients (Figures 1(g) and 1(h)).

### 3.2. LINC02389 Exerted Oncogenic Role in NSCLC Cells

**Figure 2:**
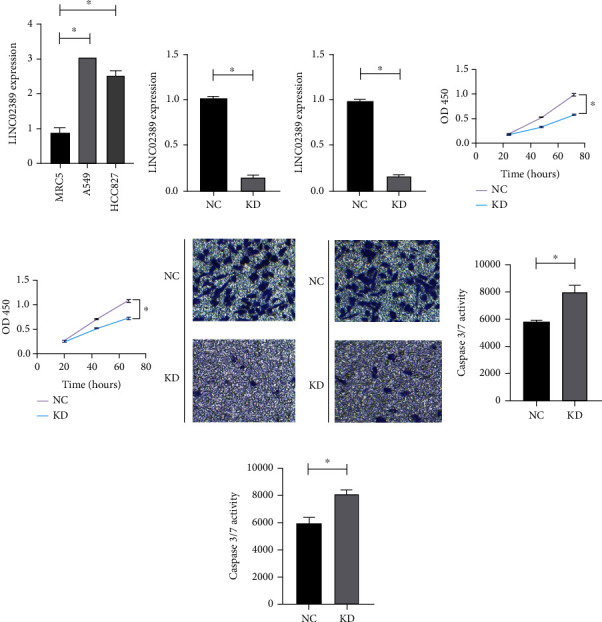
(a) LINC02389 expression in A549 and HCC827 cell lines compared with MRC5. (b) Knockdown of LINC02389 in A549. (c) Knockdown of LINC02389 in HCC827. (d) CCK-8 results for LINC02389 knockdown in A549. (e) CCK-8 results for LINC02389 knockdown in HCC827. (f) Transwell assay to detect the effect of LINC02389 knockdown on migration ability in A549. (g) Transwell assay to detect the effect of LINC02389 knockdown on migration ability in HCC827. (h) Caspase 3/7 assay to evaluate the effect of LINC02389 knockdown on apoptosis rate in A549. (i) Caspase 3/7 assay to evaluate the effect of LINC02389 knockdown on apoptosis rate in HCC827.

We have found aberrantly high expression of LINC02389 in A549 and HCC827 compared with MRC5 (Figure 2(a)). Then, we have knocked down LINC02389 in A549 (Figure 2(b)) and HCC827 (Figure 2(c)). CCK-8 assay showed that LINC02389 knockdown led to impaired cell proliferation rate (Figures 2(d) and 2(e)). Then, we have applied Transwell assay and found that cell migration assay was decreased by LINC02389 knockdown (Figures 2(f) and 2(g)). Caspase 3/7 assay indicated that LINC02389 knockdown resulted in enhanced apoptosis (Figures 2(h) and 2(i)).

### 3.3. LINC02389, Induced by H3K27ac, Regulated Cisplatin Resistance of NSCLC

**Figure 3:**
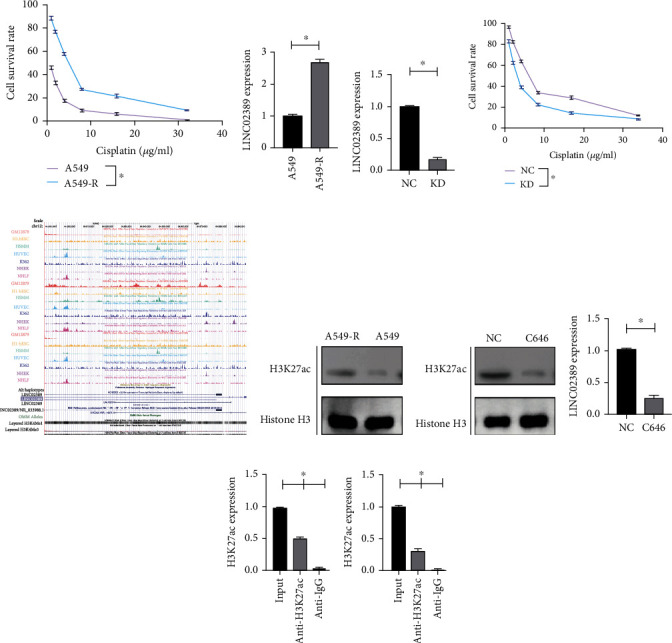
(a) CCK-8 assay to evaluate the IC50 of A549 and A549-R (cisplatin resistant) to cisplatin. (b) LINC02389 expression in A549-R compared with A549. (c) Knockdown of LINC02389 expression in A549-R. (d) CCK-8 assay to evaluate the effect of LINC02389 knockdown in IC50 of A549-R to cisplatin. (e) UCSC genome browser to explore the H3K27ac enrichment. (f) H3K27ac expression in A549-R compared with A549. (g) H3K27ac expression in A549-R treated with C646. (h) LINC02389 expression in A549-R treated with C646. (i) ChIP assay to evaluate the enrichment of H3K27ac in the promoter region of LINC02389 in A549-R. (j) ChIP assay to evaluate the enrichment of H3K27ac in the promoter region of LINC02389 in A549-R treated with C646.

We have constructed cisplatin-resistant A549 cell line (A549-R); the RI was 4.79/0.89 = 5.38 (Figure 3(a)). We found that LINC02389 was overexpressed in A549-R compared with A549 (Figure 3(b)). After knocking down LINC02389 in A549-R (Figure 3(c)), we have noticed decreased IC50 of A549-R (2.21 *μ*g/ml), suggesting that LINC02389 was essential in mediating cisplatin resistance of A549 (Figure 3(d)). Based on these results, we assumed that LINC02389 expression was activated in A549-R. According to UCSC genome browser, we found that H3K27ac was important in promoting A549 expression (Figure 3(e)). In addition, we have detected overexpression of H3K27ac in A549-R compared with A549 (Figure 3(f)). By treating with C646, we have found decreased expression of H3K27ac in A549-R (Figure 3(g)); and further, LINC02389 expression can be decreased by C646 (Figure 3(h)). Then, we have found that H3K27ac was enriched in the promoter region of LINC02389 by ChIP (Figure 3(i)), and this enrichment can be suppressed by C646 (Figure 3(j)).

### 3.4. miR-7-5p Might Be the Downstream Target of LINC02389 in Regulating Cisplatin Resistance

**Figure 4:**
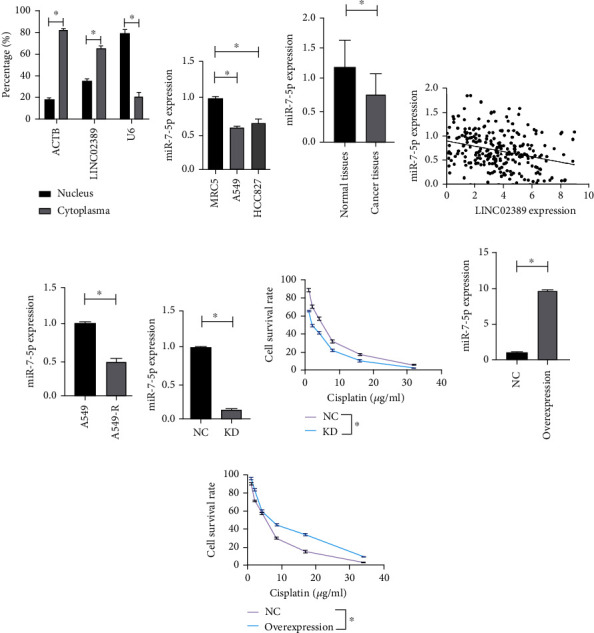
(a) LINC02389 expression in the nucleus and cytoplasma in A549-R. (b) miR-7-5p expression in A549 and HCC827 compared with MRC5. (c) miR-7-5p expression in recurrent and nonrecurrent non-small-cell lung cancer patients. (d) The correlation between miR-7-5p and LINC02389 expressions in non-small-cell lung cancer patients. (e) miR-7-5p expression in A549-R compared with A549. (f) Knockdown of miR-7-5p in A549-R. (g) CCK-8 assay to evaluate the effect of miR-7-5p knockdown in IC50 of A549-R to cisplatin. (h) Upregulation of miR-7-5p in A549-R. (i) CCK-8 assay to evaluate the effect of miR-7-5p upregulation in IC50 of A549-R to cisplatin.

We have found that LINC02389 was predominately expressed in the cytoplasm of A549-R (Figure 4(a)); therefore, we assumed that LINC02389 might regulate cisplatin resistance by sponging microRNAs. Based on starBase, we have predicted potential microRNA targets for LINC02389. By qRT-PCR, we have found that only miR-7-5p was significantly downregulated in NSCLC cells (Figure 4(b)). Moreover, in NSCLC patients, we have found that miR-7-5p was downregulated in NSCLC patients (Figure 4(c)) and in LINC02389 high expressed patients as well (Figure 4(d)). In addition, miR-7-5p was suppressed in A549-R (Figure 4(e)). Therefore, we assumed that LINC02389 might sponge miR-7-5p to regulate cisplatin resistance.

In A549-R, we have downregulated the expression of miR-7-5p (Figure 4(f)) and found that IC50 of A549-R was impaired (2.16 *μ*g/ml) (Figure 4(g)); however, enhanced IC50 of A549-R was detected after miR-7-5p overexpressed (6.36 *μ*g/ml) (Figures 4(h) and 4(i)).

### 3.5. LINC02389 Interacted with miR-7-5p to Regulate Cisplatin Resistance

**Figure 5:**
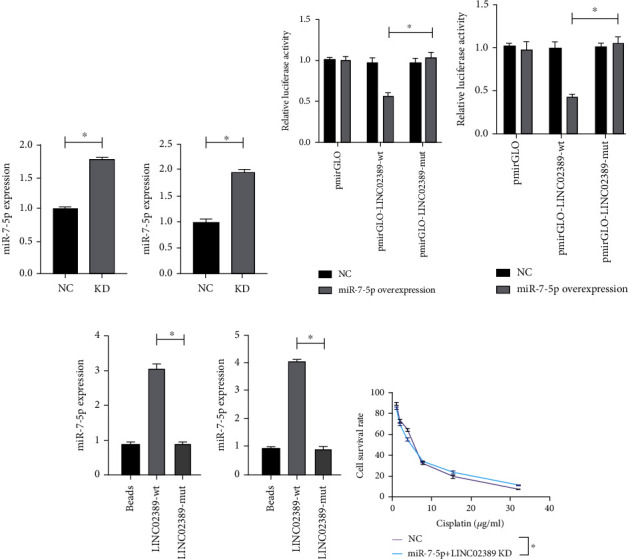
(a) miR-7-5p expression in LINC02389 knocked down A549 cell line. (b) miR-7-5p expression in LINC02389 knocked down HCC827 cell line. (c) Dual luciferase activity assay to test the binding of LINC02389 to miR-7-5p in A549. (d) Dual luciferase activity assay to test the binding of LINC02389 to miR-7-5p in HCC827. (e) RNA pull-down assay to test the interaction between miR-7-5p and LINC02389 in A549. (f) RNA pull-down assay to test the interaction between miR-7-5p and LINC02389 in HCC827. (g) miR-7-5p knockdown could reverse the effect of LINC02389 knockdown on IC50 of A549-R to cisplatin.

After knocking down LINC02389, we have found aberrantly high expression of miR-7-5p in A549 and A549-R (Figures 5(a) and 5(b)). Then, dual luciferase reporter gene assay was applied and showed that miR-7-5p might interact with LINC02389 in A549 and A549-R (Figures 5(c) and 5(d)). Then, RNA pull-down assay confirmed the crosstalk between LINC02389 and miR-7-5p in A549 and A549-R (Figures 5(e) and 5(f)). Then, we knocked down LINC02389 and miR-7-5p simultaneously and found that IC50 was not significantly influenced compared with the control group (Figure 5(g)).

### 3.6. LINC02389/miR-7-5p Regulated Cisplatin Resistance by Targeting Oxidative Stress

**Figure 6:**
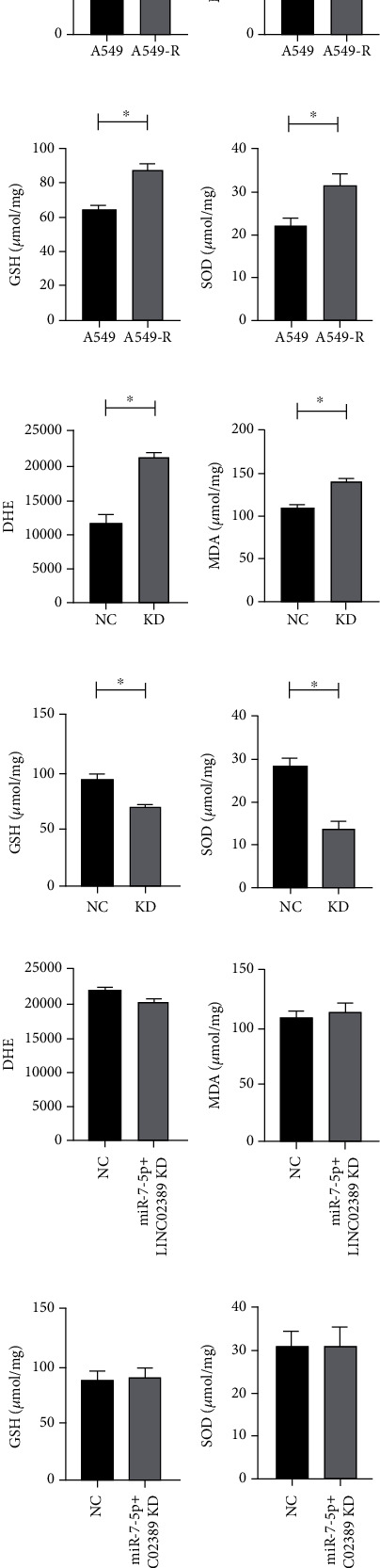
(a–d) DHE, MDA, GSH, and SOD expressions in A549-R compared with A549. (e–h) DHE, MDA, GSH, and SOD expressions in LINC02389 knocked down A549-R. (i–l) DHE, MDA, GSH, and SOD expressions in LINC02389 and miR-7-5p knocked down A549-R.

Previously, we have found that oxidative stress was alleviated in cisplatin-based chemotherapy-resistant patients. In this part, we have detected decreased ROS and MDA and increased GSH and SOD in A549-R compared with A549 (Figures 6(a)–6(d)). Then, we found that by knocking down LINC02389 in A549-R, ROS and MDA were increased and GSH and SOD were decreased (Figures 6(e)–6(h)). Moreover, we knocked down LINC02389 and miR-7-5p simultaneously and found that ROS, MDA, GSH, and SOD levels were not significantly influenced (Figures 6(i)–6(l)).

## 4. Discussion

The mechanism by which chemotherapy induces drug resistance in lung cancer is not fully understood. At present, a variety of mechanism theories have been formed, such as DNA damage repair, cancer stem cells, antiapoptosis, and immune escape [29]. However, various molecular mechanisms need to act by affecting RNA or protein expression. miR-7-5p has been extensively studied in NSCLC due to its tumor suppressive effect, and it can affect cell proliferation and apoptosis [28, 30, 31]. LINC02389 has also been suggested to play an important role in NSCLC in previous studies, but its specific mechanism is unclear. We performed qRT-PCR and Western blot analysis of NSCLC and its adjacent tissues. The results showed that LINC02389 expression was higher in NSCLC cell, whereas miR-7-5p was the opposite. Besides, the expression of LINC02389 was significantly reduced in local recurrence patients. These confirm that LINC02389 and miR-7-5p are indeed differentially expressed in NSCLC. We further explored their effects on proliferation and apoptosis in NSCLC cell lines. Knockdown of LINC02389 inhibited apoptosis by CCK-8 and flow cytometry assays, whereas knockdown of miR-7-5p showed the opposite effect. This is similar to the study by Li [28]. In addition, we also found that both LINC02389 and miR-7-5p can promote cancer cell proliferation. In subsequent cisplatin-resistant lung cancer cells, we found that LINC02389 was overexpressed and miR-7-5p was suppressed. Meanwhile, only miR-7-5p was significantly downregulated in cisplatin-resistant cells by qRT-PCR.

The effects of LINC02389 and miR-7-5p on tumor cells were significantly different. This change in the drug-resistant cancer cells was induced in vitro and appears to be related. Therefore, we further verified the relationship between them. Firstly, we found that in patients with high LINC02389 expression, miR-7-5p expression was low, and they were negatively correlated. In contrast, the expression of miR-7-5p was significantly increased after LINC02389 knockdown. Subsequently, we used the dual-luciferase reporter gene assay to determine the negative correlation between LINC02389 and miR-7-5p in NSCLC cancer cells in vitro, and RIP and RNA pull-down assays suggested their binding activity. In other words, LINC02389 can act as a ceRNA to reduce the expression of miR-7-5p. We elucidate the expression of LINC02389 and miR-7-5p in cisplatin-resistant cells. Furthermore, the inhibitory effect of cisplatin was reversed when LINC02389 was overexpressed in NSCLC cells. And this reversal could be attenuated by the expression of miR-7-5p. This implies that LINC02389 promotes drug resistance by downregulating miR-7-5p. Our study is the first to demonstrate the role of LINC02389 as an upstream regulator in the downregulation of miR-7-5p.

Oxidative stress is an imbalance of free radicals and their metabolism [32]. It causes DNA damage in nucleotides [33]. Many signaling pathways can trigger signaling cascades leading to ROS, such as the NF-*κ*B pathway [34]. In recent years, oxidative stress has also been considered a possible underlying mechanism for tumor cells to develop chemoresistance. We observed significant differences in the expression of MDA and GSH in local recurrence patients. In in vitro experiments, we investigated the expression of many ROS biomarkers at the protein and RNA levels. We found that ROS and MDA were reduced and GSH and SOD were increased in cisplatin-resistant cells, which were reversed by the knockout of LINC02389. However, when LINC02389 and miR-7-5p were simultaneously knocked out, we did not observe significant changes in any of all oxidative stress indicators. This suggests that LINC02389 can promote oxidative stress in cisplatin-resistant cells, and this promotion is achieved by regulating the expression of miR-7-5p.

## Data Availability

All authors ensure that data and materials are available and transparent.
